# Disrupting redox stabilizer: a novel therapeutic strategy for colorectal cancer

**DOI:** 10.1186/s40880-019-0355-y

**Published:** 2019-03-12

**Authors:** Dunyaporn Trachootham, Watcharapol Khoonin

**Affiliations:** 1 Institute of Nutrition, Mahidol University, 999 Phutthamonthon Sai 4 Road, Salaya, Nakhon Pathom 73170 Thailand; 2 Chulabhorn Oncology Medical Center, Chulabhorn Hospital, Bangkok, 10210 Thailand

Altered stress response and metabolic reprogramming have been recently proposed as emerging hallmarks of cancer cells [1]. These tumorigenic properties provide survival and proliferative advantages under various stressed-conditions [2]. Several lines of evidences suggest that cancer cells exhibited increased reactive oxygen species (ROS) and are under oxidative stress [3]. To counterbalance the stress and survive, cancer cells are dependent on ROS scavenging systems, especially glutathione. Inhibiting such ROS scavenging system had proven effective in shifting the redox balance toward an increase in oxidative stress, leading to selective killing of cancer cells [3].

As depicted in Fig. 1, maintaining homeostasis between ROS producing and scavenging requires reduced glutathione (GSH), the most abundant tripeptide in normal cells. In order to reuse glutathione, the recycling of oxidized glutathione (GSSG) back to reduced GSH requires reducing equivalent nicotinamide adenine dinucleotide phosphate (NADPH). Enzymes generating NADPH include G-6-PD, malic enzyme 1 and 2, and methylenetetrahydrofolate dehydrogenase 1/2 (MTHFDH 1/2) [4]. These NADPH generating enzymes play important roles in stabilizing redox homeostasis, thereby, promoting cell survival under stressed condition. Convincing evidences suggest that certain type of cancer cells can stimulate the overexpression of these NADPH producing enzymes, as a result of metabolic switching mechanism [5]. This in turn leads to an increase in ROS which can promote genomic instability, causing altered signal transduction, and sustain tumor formation and growth.

**Fig. 1:**
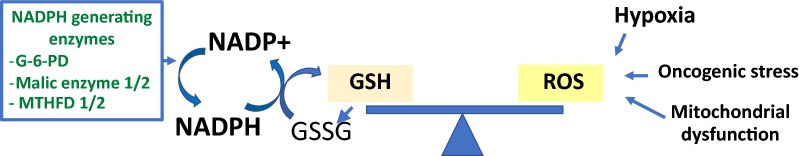
Redox homeostasis and the role of NADPH. NADPH, nicotinamide adenine dinucleotide phosphate; NADP+, triphosphopyridine nucleotide; G-6-PD, glucose-6-phosphate dehydrogenase; MTHFD 1/2, methylenetetrahydrofolate dehydrogenase 1/2; GSH, reduced glutathione; GSSG, oxidized glutathione; ROS, reactive oxygen species

Targeting such NADPH generators may serve as a novel therapeutic strategy to selectively disrupt redox homeostasis and induce ROS-mediated cell death [3]. This concept was proven experimentally in a recently published article in the *Journal of National Cancer Institute*, entitled “Modulation of redox homeostasis by inhibition of MTHFD2 in colorectal cancer: mechanisms and therapeutic implications” by Ju et al. [6]. Upon investigating the expression profiling of various potential NADPH-producing enzymes, the researchers observed a significant and prominent overexpression of MTHFD2 in colorectal cancer tissues, compared with adjacent normal tissues, through data mining from tissue microarray and The Cancer Genome Atlas (TCGA). Importantly, colorectal cancer patients with high expression of MTHFD2 had a shorter overall survival and disease-free survival than patients with low MTHFD2 expression. Using in vitro and in vivo experiments, they further showed that the inhibition of MTHFD2, either by genetically knocking-down or chemically inhibition using folate analog LY345899 as an MTHFD2 inhibitor, could disturb the NADPH and redox homeostases and accelerate cell death under oxidative stress, such as hypoxia, or causing in vitro anchorage independence and in vivo impaired tumor growth and metastasis. Their results provide solid evidence that targeting redox stabilizers, such as MTHFD2, could be a potential therapeutic strategy for treating cancer.

The one-carbon metabolism is a complex process which supports DNA synthesis through the generation of purine and thymidine, regulates methylation through the generation of *S*-adenosyl methionine (SAM), and provides NADPH for the lipid metabolism and redox homeostasis maintenance [7]. These events eventually support multiple physiological processes such as cell proliferation, gene expression, and cell survival [7]. MTHFD2 has been found as being an important enzyme in the metabolic process of one-carbon amino acid metabolism [8]. MTHFD2 can catalyze the conversion of 5,10-methylene tetrahydrofolate to 10-formyl tetrahydrofolate in the mitochondria to generate NADPH as a byproduct [8]. Thus, it is highly possible that the inhibition of MTHFD2 not only could perturb redox homeostasis but also may disrupt the one-carbon metabolism. In fact, recent studies had revealed that the inhibition of folate pathway could induce oxidative stress and suppress cancer progression [9]. This is in line with other reports showing the tumor-promoting effect of folate supplement in breast cancer and colon cancer [10]. Taken together, targeting MTHFD2 may attack multiple hallmarks of cancer and likely provide therapeutic benefit.

During different stressed conditions, such as anchorage-independent growth and hypoxia, cancer cells are known to adopt a metabolic switch for energy supply and NADPH production, necessary for their rapid proliferation and ROS detoxification. Although oncogenic signal plays an important role in metabolic reprogramming and oxidative stress, the underlying mechanism is poorly understood. Interestingly, the study by Ju et al. [6] showed that Kras induces c-Myc stabilization by activating PI3K/Akt and ERK pathways, while c-Myc alters tumor metabolism by up-regulating MTHFD2 expression to promote colorectal cancer progression. Thus, the study uncovers a mechanistic explanation for the link between oncogenic signals and the metabolic adaptation machineries driving colorectal cancer development. Disruption of such connectors may provide a basis for cancer prevention; thereby, warrant further studies.

Several clinically available anti-folate drugs, such as methotrexate, fluorouracil (5-FU) and gemcitabine, were known to be competitive inhibitor of folate. However, those drugs do not specifically target cancer cells, thereby, resulting in several side effects. Uniquely, MTHFD2 is predominantly expressed during embryonic development but is lowly expressed in normal human adult cells. To eradicate cancer cells via MTHFD2 suppression while sparing healthy cells has become possible. Moreover, recent progress has shed new light on the development of selective inhibitors of MTHFD2. A recent study reported that LY345899, the first substrate-based inhibitor of MTHFD2, could suppress the MTHFD enzyme in the cytoplasm and mitochondria. Importantly, Ju et al. [6] showed the potential in vivo antitumor activity of LY345899, validated it in patients’-derived subcutaneous xenograft and mesenteric metastatic mouse models and found that neither weight loss nor other signs of acute or delayed toxicity were observed during the prescribed treatment. These findings suggest that the possible clinical implications of LY345899 deserve further investigations as an anti-cancer therapy (Fig. 2). Specifically, as redox disturbance underlies the therapeutic effects of several chemotherapeutic drugs, exploring the potential combinational effects and structural optimization of LY345899 would be a direction worth heading to in the nearing future.

**Fig. 2:**
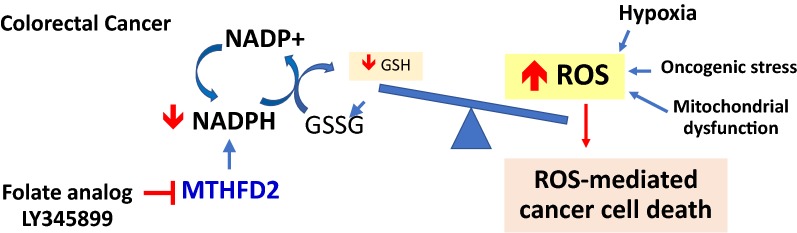
Disrupting the redox homeostasis by inhibition of NADPH-producing enzyme such as MTHFD2 as a novel therapeutic strategy for colorectal cancer. Abbreviations: NADPH, nicotinamide adenine dinucleotide phosphate; NADP+, triphosphopyridine nucleotide; G-6-PD, glucose-6-phosphate dehydrogenase; MTHFD 2, methylenetetrahydrofolate dehydrogenase 2; GSH, reduced glutathione; GSSG, oxidized glutathione; ROS, reactive oxygen species

In conclusion, Ju et al. provided relevant proofs of the concept that disrupting redox stabilizers, such as MTHFD2, could be a novel therapeutic strategy. However, owing to the complex function of one-carbon metabolism, further investigation on the clinical implications of the antitumor activity of LY345899 is warranted for monitoring the efficacy and side effects of using such strategy in the treatment of colorectal cancer patients.

## Abbreviations

ROS: reactive oxygen species
G-6-PD: glucose-6-phosphate dehydrogenase
MTHFD1: methylenetetrahydrofolate dehydrogenase 1
MTHFD2: methylenetetrahydrofolate dehydrogenase 2
NADP+: triphosphopyridine nucleotide
NADPH: nicotinamide adenine dinucleotide phosphate
GSH: reduced glutathione
GSSG: oxidized glutathione
TCGA: The Cancer Genome Atlas
SAM: *S*-adenosyl methionine
5-FU: fluorouracil
MTHFD ½: methylenetetrahydrofolate dehydrogenase 1/2
